# miR-193a-3p Promotes the Invasion, Migration, and Mesenchymal Transition in Glioma through Regulating BTRC

**DOI:** 10.1155/2021/8928509

**Published:** 2021-02-09

**Authors:** Dan-Dan Zhou, Hong-Li Li, Wei Liu, Li-Ping Zhang, Quan Zheng, Jun Bai, Ya-Qiong Hu, Chong-Gao Yin, Shi-Jun Lv, Bao-Gang Zhang

**Affiliations:** ^1^Department of Pathology, School of Clinical Medicine, Weifang Medical University, Weifang, Shandong 261053, China; ^2^Experimental Center for Medical Research, Weifang Medical University, Weifang, Shandong 261053, China; ^3^School of Clinical Medicine, Weifang Medical University, Weifang, Shandong 261053, China; ^4^College of Nursing, Weifang Medical University, Weifang, Shandong 261053, China

## Abstract

**Background:**

The present study is aimed at exploring the specific expression of miR-193a-3p and the mechanism underlying miR-193a-3p-mediated mesenchymal transition (MT), invasion, and migration in glioma.

**Methods:**

The gene expression profile datasets of GSE39486 and GSE25676 were downloaded from the National Center for Biotechnology (NCBI). Data regarding the expression of miR-193a-3p and survival curves were derived from Chinese Glioma Genome Atlas (CGGA). Online websites including miRWalk, DIANA, and starbase were employed to predict the target genes for miR-193a-3p. The Gene Ontology (GO) and Kyoto Encyclopedia of Genes and Genomes (KEGG) pathway enrichment analyses were performed by the Omicsbean online software. Module analysis of the protein-protein interaction (PPI) networks was performed by the plug-in Molecular Complex Detection (MCODE), and the degrees of genes were calculated by CytoHubba plug-in of Cytoscape. Survival curves were based on the Gene Expression Profile Interaction Analysis (GEPIA). Transwell, wound healing, and Western blot experiments were performed to investigate the effects of miR-193a-3p and beta-transducin repeat containing E3 ubiquitin protein ligase (BTRC) on the invasion, migration, and MT of glioma.

**Results:**

miR-193a-3p was highly expressed in glioma tissues and significantly correlated with poor survival in patients with glioma. The target genes for miR-193a-3p were involved in many cancer-related signaling pathways. The PPI showed 11 genes with both high degrees and MCODE scores in the network. Survival analysis demonstrated that the expression of BTRC was significantly correlated with the prognosis of patients with glioma. The results from the transwell, wound healing, and Western blot analyses suggested that miR-193a-3p promoted the invasion, migration, and MT of glioma cells, which could be reversed by BTRC.

**Conclusions:**

miR-193a-3p was upregulated in patients with glioma and could affect the invasion, migration, and MT of glioma by regulating BTRC.

## 1. Introduction

Glioma is the most common malignancy in the central nervous system [1] and possesses poor prognosis and high recurrence rate [2]. Despite the rapid advances in surgery, chemotherapy, and radiation therapy during the last decades, the average survival time for patients with malignant glioma has not been improved significantly [3]. Therefore, it is to investigate the prognostic biomarkers and find new treatments for glioma.

MicroRNAs (miRNAs) are a family of small noncoding RNAs with mature forms of approximately 18-22 nucleotides [4]. They can target the 3′ translation region of messenger RNA (mRNA) and then trigger the degradation or inhibit the translation of the latter [5]. Abnormal expression and regulation of miRNAs in glioma have been extensively studied [6]. miRNA levels in cerebrospinal fluid and brain tissues from patients with glioma are reliable tumor markers and may have diagnostic value for glioma [7, 8]. It has been confirmed that the miR-193a-3p in exosomes promotes lung cancer cell invasion by activating STAT3 signaling-induced epithelial-mesenchymal transition (EMT) and suppresses the progression of non-small-cell lung cancer via the p53/Slug/L1CAM pathway [9]. Beta-transducin repeat containing E3 ubiquitin protein ligase (BTRC) is a member of the F-box and WD40 repeat family of proteins and associated with EMT-related protein degradation [10]. Other scholars have proposed that BTRC promotes the metastasis of esophageal squamous cell carcinoma by activating NF-*κ*B signaling pathway and can modulate cell cycle and different signal transduction pathways by ubiquitination, which is the key to carcinogenesis [11, 12]. BTRC has also been demonstrated a vital role in the EMT process since BTRC mediates the snail's ubiquitination in cancer and the former's inhibition leads to the upregulation of snails, which induces EMT [13]. However, whether miR-193a-3p is involved in the invasion and migration of glioma by binding to BTRC remains poorly understood.

This study is aimed at exploring the glioma miRNA profiling by array data from the GEO databases and miRNA-mRNA interactions in glioma through bioinformatics analyses and experimental validation. Here, we examined the biological effects of increased miR-193a-3p on glioma. Based on the online tools, mRNAs that were likely to bind to miR-193a-3p were predicted. Subsequently, we established a PPI network and corroborated that BTRC played a pivotal role in the PPI network and was closely related to cancer. Finally, by extensive bioinformatics analyses, we confirm that upregulated miR-193a-3p serves as a key regulator of BTRC. Upregulated miR-193a-3p or downregulated BTRC can significantly promote the invasion, migration, and MT of glioma cell lines. Taken together, our results demonstrate that miR-193a-3p and BTRC may serve as a promising therapeutic target for glioma.

## 2. Materials and Methods

### 2.1. Microarray Data

The miRNA and mRNA gene chip datasets for glioma, GSE39486 and GSE26576, were retrieved and downloaded from the GEO database (https://www.ncbi.nlm.nih.gov/geo/), respectively. GSE39486 dataset included 3 normal fetal brains and 3 glioma tissues. GSE26576 included 35 glioma tissues and 2 normal tissues.

### 2.2. Screening of Differentially Expressed miRNAs and Prediction of miRNA Target Genes

The GEO2R was employed to screen miRNAs differentially expressed in normal fetal brain and glioma tissues. *P* value ≤0.05 and ∣log2 FC | ≥1 was used as thresholds. The selected miRNAs were employed to the heatmap and volcano plot by using the R studio ggplot2 package. The target genes were predicted through the online prediction websites of miRWalk (http://mirwalk.umm.uni-heidelberg.de/), DIANA (http://diana.imis.athena-innovation.gr/DianaTools/index.php?r=site/index), and starbase (http://starb-ase.sysu.edu.cn/).

### 2.3. Cell Culture

Four human glioma cell lines, namely, U251, U87, LN-229, and H4, were obtained from the American Type Culture Collection (ATCC) (Manassas, VA, USA). All of them were cultured in RPMI 1640 medium (HyClone, SH30809.01B) supplemented with 10% fetal bovine serum (FBS) (HyClone, SH30070.03) at 37°C with 5% CO_2_.

### 2.4. Construction and Analysis of PPI Network

The online website of STRING (https://string-db.org/) was used to construct a PPI network [14] and export the TSV format data which was then imported into Cytoscape 3.7.1 [15]. The Molecular Complex Detection (MCODE version 1.4.2) application (http://apps.cytoscape.org/apps/mcode) was utilized to select the key modules from the PPI network in Cytoscape with MCODE scores > 5, with the independent nodes deleted. The subnetwork was visualized with a plug-in of CytoHubba in Cytoscape which was used to identify the degrees of genes in the PPI network, with degree ≥ 10 as the cut-off.

### 2.5. Functional Enrichment Analysis of Target Genes

GO annotation and KEGG pathway analyses of the target genes were carried out using Omicsbean (http://www.omicsbean.cn/report/proteomics/328682/). *P* < 0.05 was considered statistically significant.

### 2.6. The Online Public Database

Chinese Glioma Genome Atlas (CGGA) can be used to explore brain tumor datasets from more than 2000 samples from China. The glioma microRNA expression profiles and survival analyses from CGGA were downloaded. The GEPIA (http://gepia.cancer-pku.cn/) database provides interactive analyses of genes for cancer and normal tissues [16]. To further elucidate the relationship between the expression of key genes and prognosis in patients with glioma, key gene survival and statistical analyses using log rank were downloaded from GEPIA, and *P* < 0.05 was considered statistically significant.

### 2.7. RNA Extraction and Quantitative Real-Time PCR (qRT-PCR)

RNA extraction and qRT-PCR were performed as described previously [17]. Expression of miR-193a-3p was normalized to that of U6 using the relative threshold cycle method, and then converted to fold changes. The primers used in the present work were as follows: miR-193a-3p RT primer: GTCGTATCCAGTGCAGGGTCCGAGGTATTCGCACTGGATACGACACTGGG; miR-193a-3p forward: 5′-CGCGAACTGGCCTACAAAGTG-3′, reverse: 5′-AGTGCAGGGTCCGAGGTATT-3′; U6 forward: 5′-CTCGCTTCGGCAGCACA-3′, reverse: 5′-AACGCTTCACGAATTTGCGT-3′.

### 2.8. Transfection of Glioma Cells

LN-229 cells (2 × 10^5^) were seeded in six-well plates and incubated at 37°C with 5% CO_2_ overnight. Lipofectamine 2000 was used for transfection (Invitrogen; Thermo Fisher Scientific, Carlsbad, CA, USA) according to the manufacturer's instructions. miR-193a-3p mimics, miR-193a-3p inhibitor, BTRC siRNAs (si-BTRC), and corresponding negative control (miR-NC and si-NC) were purchased from GeneChem company (Shanghai, China). Stable transfected cells were maintained within G418 of 300 *μ*g/mL for 14 days. Transfection efficiency was evaluated by qRT-PCR.

### 2.9. Transwell Assay

LN-229 cells (1 × 10^5^) were seeded in the upper chamber within serum-free medium, with 1640 medium supplemented with 10% FBS in the lower one. The bottom of the chamber was precoated with Matrigel. The cells were incubated for 24 h at 37°C. Then, the membranes of the cells were fixed and stained with Giemsa followed by being observed under a microscope. Three fields of view were randomly selected to calculate the average number of cells.

### 2.10. Dual Luciferase Reporter Assay

Both wild type (WT) and mutant (MUT) 3′UTRs of BTRC mRNA were cloned into a luciferase reporter vector (psi-CHECK-2, Promega, WI, USA). 293T cells were cotransfected with the miR-193a-3p mimics and BTRC-WT or BTRC-MUT vector. After 48 h, luciferase and renilla signals were measured using a dual luciferase assay system (Promega, WI, USA).

### 2.11. Wound-Healing Assay

Wound-healing assay was performed as described previously [18]. In brief, LN-229 cells were cultured in 6-well plates. After the cells had grown to 90% confluence, three parallel scratch wounds were made on each well with a 10 *μ*L pipette tip. The culture was continued using fresh medium containing 1% FBS. Photos were taken at 0 and 24 hours. All assays were performed independently in triplicate.

### 2.12. Western Blot

Total proteins were extracted from glioma cells using radio immunoprecipitation assay (RIPA) lysis buffer. The protein samples were separated by sodium dodecyl sulfate-polyacrylamide gel electrophoresis (SDS-PAGE) and transferred onto polyvinylidene difluoride membranes (PVDF) (Millipore Inc, Billerica, MA, USA). The membrane was then blocked with 5% defatted milk in PBST (PBS containing 0.1% Tween 20) for 1 h and then subjected to the following antibodies: anti-BTRC monoclonal antibody (EnoGene, 1 : 500), anti-N-cadherin rabbit polyclonal antibody (Cell Signaling Technology, CST, 1 : 500), anti-T-cadherin rabbit polyclonal antibody (CST, 1 : 500), anti-vimentin rabbit polyclonal antibody (CST, 1 : 1000), or anti-*β*-actin rabbit polyclonal antibody (CST, 1 : 2000). After being rinsed three times with cold PBS, the membrane was incubated with HRP-conjugated anti-mouse IgG or anti-rabbit IgG antibodies (CST, 1 : 5000) for 2 h at room temperature. Immunoreactive bands were visualized using an enhanced chemiluminescence (ECL) kit (Beyotime Biotechnology, Shanghai, China). The gray values were analyzed using the Image J v1.8.0 software (Image J Software, Bethesda, USA), with *β*-actin as the internal reference gene.

### 2.13. Human Protein Atlas (HPA) Datasets

Based on the high specificity of antibody binding to antigen, immunohistochemistry can reveal the relative distribution and abundance of proteins. The HPA (https://www.proteinatlas.org) is the world's largest and most comprehensive database for human tissue cell protein spatial distribution [19], with numerous features and potential application prospects. We employed HPA to investigate the differences in BTRC expression between normal and glioma tissues.

### 2.14. Statistical Analyses

Differences among groups were assessed by paired, two-tailed Student's *t*-test. A value of *P* < 0.05 was considered statistically significant. All statistical analyses were performed using the GraphPad Prism 5 software (Graph-Pad Software, San Diego, USA).

## 3. Results

### 3.1. miR-193a-3p Was Upregulated in Glioma and Associated with Poor Prognosis in Patients with Glioma

**Figure 1:**
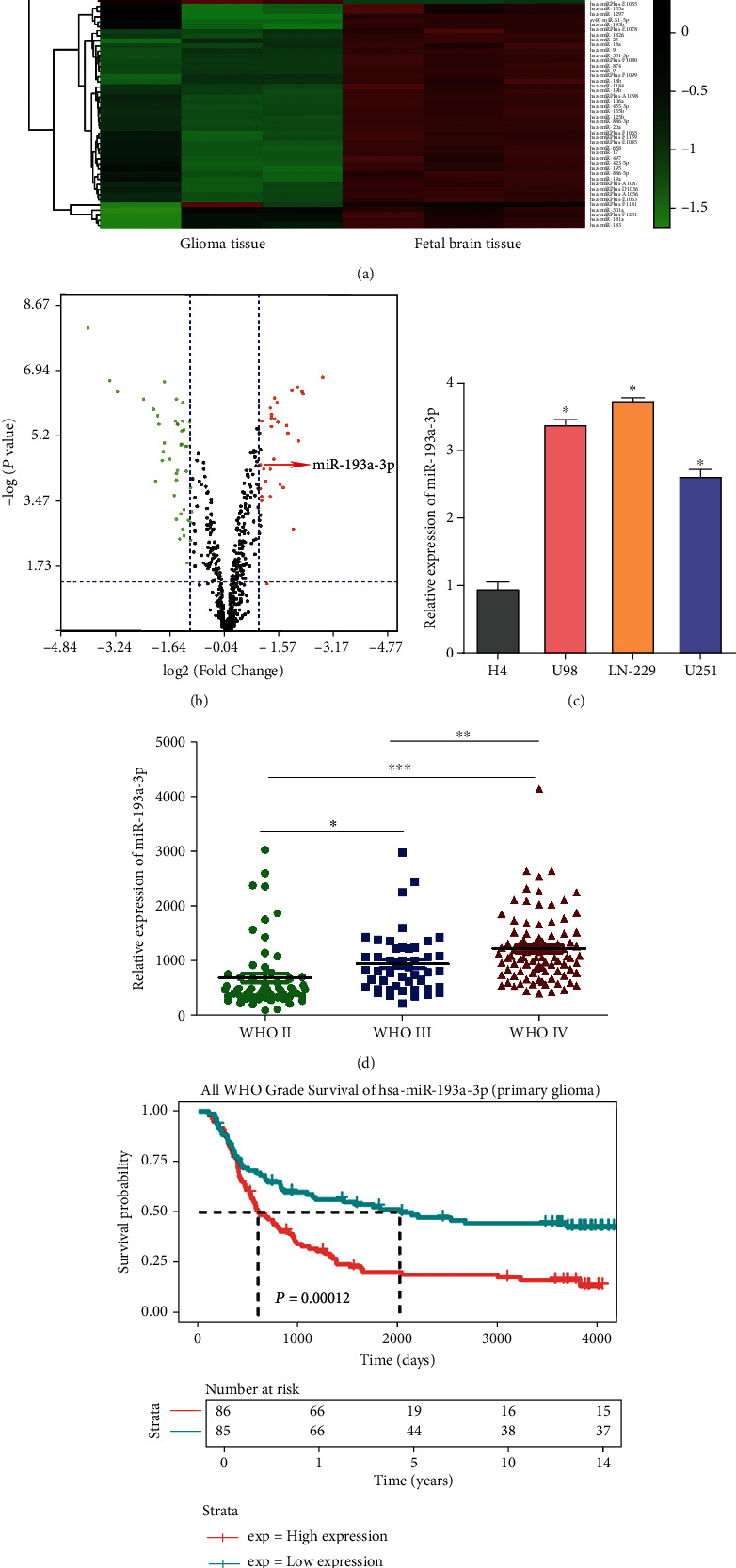
High expression of miR-193a-3p was observed in glioma. (a) Hierarchical clustering heatmap of differential expression miRNA analyses in GSE39486. (b) Volcano plot. Red and green indicate up- and downregulated miRNA in glioma, respectively. (c) Expression of miR-193a-3p in glioma cell lines determined by qRT-PCR. U6 was used as control for normalization. Data are shown as the means ± SD based on three independent experiments. ^∗^*P* < 0.05 versus the cell line of H4. (d) Expression level of miR-193a-3p in glioma tissues with WHO stages II, III, and IV. ^∗^*P* < 0.05, ^∗∗^*P* < 0.01, and ^∗∗∗^*P* < 0.001. (e) Kaplan–Meier survival plots based on the expression of miR-193a-3p in patients with primary glioma (*n* = 171 and *P* < 0.05).

The GSE39486 dataset was downloaded from the GEO database, and Table 1 displays the characteristic information for the sample. Differential expression analysis for GSE39486 showed 33 upregulated miRNAs and 44 downregulated miRNAs in glioma tissues, compared with normal fetal brain ones (Figures 1(a) and 1(b)). Then, we examined the expression of miR-193a-3p in glioma cell lines by qRT-PCR and found higher expression of miR-193a-3p in three high-grade glioma cell lines of U87, U251, and LN-229 than in the low-grade one of H4 (Figure 1(c)). LN-229 cell lines were selected for subsequent experiments due to the highest expression of miR-193a-3p among the three cell lines employed. The relationship between miR-193a-3p expression and glioma staging based on World Health Organization (WHO) staging system was investigated through the CGGA public database. The expression of miR-193a-3p was higher in stages III and IV than that in stage II (Figure 1(d)). The survival curves of miR-193a-3p were also predicted by CGGA. The clinical characteristic of CGGA patients is shown in Table 2. Of all 171 patients enrolled, the 1-year survival rate was 76.7%, the 5-year survival rate was 22%, the 10-year survival rate was 18.6%, and the 14-year survival rate was 17.5% in high miR-193a-3p expression groups. The 1-year survival rate of the low miR-193a-3p expression was 77.6%, the 5-year survival rate was 51.8%, the 10-year survival rate was 44.7%, and the 14-year survival rate was 43.5%. The results suggested that glioma patients with high level of miR-193a-3p demonstrated significantly lower survival rate (*P* < 0.05) (Figure 1(e)). Therefore, our results indicated that miR-193a-3p was highly expressed in glioma and significantly correlated with the prognosis for patients with glioma.

### 3.2. Bioinformatics Analyses and Screening of Differentially Expressed Genes

**Figure 2:**
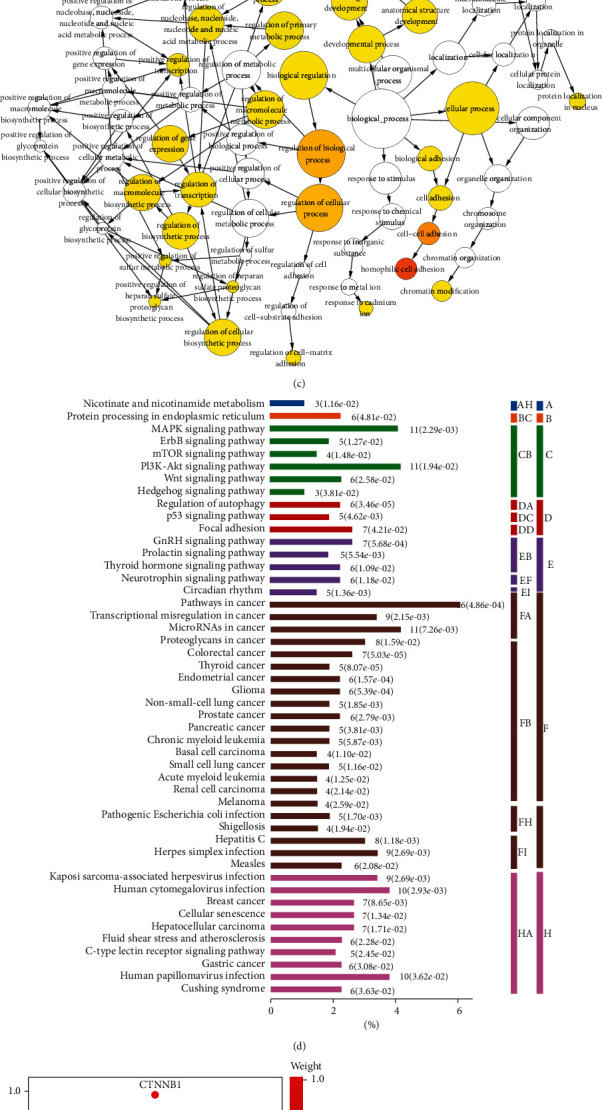
Prediction and functional annotation of target genes for miR-193a-3p. The GO analysis of (a) molecular functions, (b) cellular components, and (c) biological processes. (d) KEGG analysis of differentially expressed genes. *P* < 0.05. (e) Distribution of CTNNB1, CDK6, TSC2, BTRC, and RAP1A in KEGG pathway based on genome background enrichment. (f) The first 19 genes were chosen using CytoHubba plug-in. The more forward ranking is represented by a redder color. (g) Top 1 module from the PPI network. The more forward ranking is represented by a redder color. Abbreviations: A: metabolism; AH: metabolism of cofactors and vitamins; B: genetic information processing; BC: folding, sorting, and degradation; C: environmental information processing; CB: signal transduction; D: cellular processes; DA: transport and catabolism; DC: cell growth and death; DD: cellular community; E: organismal systems; EB: endocrine system; EF: nervous system; El: environmental adaptation; F: human diseases; FA: cancers: overview; FB: cancers: specific types; FH: infectious diseases: bacterial; FI: infectious diseases: viral; H: other and unknow; HA: other and unknow.

A total of 13695 target genes for miR-193a-3p were predicted by miRWalk, with 545 ones predicted by starbase and 509 by DIANA tools. 269 target genes were obtained from the intersection of the three databases (Supplement Figure 1A). The GO enrichment analysis was composed of three parts: biological process, cellular component, and molecular function. GO network analyses were analyzed by Cytoscape BiNGO plug-in to predict the functions of 269 target genes of miR-193a-3p. Each circle represents a GO term. The color is colored according to the enrichment degree. The darker the color is, the higher the degree of enrichment is. The direction of the arrow indicates the hierarchy. We found that binding, protein binding, ion binding, cation binding, and metal ion binding were remarkably regulated by target host genes in molecular function (Figure 2(a)). Cell part, cell, intracellular, intracellular part, organelle, and intracellular organelle were also significantly controlled by these host genes in cellular component (Figures 2(b)). The molecular function was mainly enriched in cellular process, biological regulation, regulation of biological process, regulation of cellular process, regulation of metabolic process, multicellular organismal process, regulation of cellular metabolic process, regulation of macromolecule metabolic process, regulation of primary metabolic process, and developmental process (Figure 2(c)). To investigate the roles of these target genes in signaling pathways, KEGG pathway analyses for them were performed using the Omicsbean online database. The results revealed that these target genes were mainly enriched in regulation of autophagy in colorectal cancer, breast cancer, thyroid cancer, glioma, and endometrial cancer, along with the miRNAs, transcriptional misregulation proteoglycans, and signaling pathways including MAPK and GnRH in cancer (Figure 2(d)). There were 20 genes (such as CTNNB1, CDK6, TSC2, BTRC, and RAP1A) mainly implicated in the KEGG pathway, which was based on genome background enrichment (Figure 2(e)). The STRING database was employed to collect, score, and integrate all publicly available PPI information and supplement it by calculating predictions. The goal was to achieve a comprehensive and objective global network, including physical and functional interactions [20]. In this study, a PPI network of 269 target genes was constructed using the STRING 11.1. The results showed that the network consisted of 175 nodes and 428 edges, with 94 of the 269 target genes excluded in the 175 nodes (Supplement Figure 1B). For a node, genes with high degrees of connectivity tended to be at the center of PPI network [14]. Among the 175 nodes, 19 proteins were screened by degree ≥ 10 through Cytoscape CytoHubba plug-in, namely, hub genes (CTNNB1, CCND1, KRAS, UBA52, MDM2, MAPK8, RAP1A, BTRC, PTK2, GART, MCL1, KMT2A, SRSF2, DDX5, PTCH1, CDK6, LEF1, TSC2, and H2AFJ) (Figure 2(f)). Further, another Cytoscape plug-in MCODE was layered on the PPI to identify subnetworks. Using the MCODE for cluster analysis, top 1 significant module was obtained after clustering, including MAPK8, PTCH1, MYCN, YWHAZ, UBA52, CDK6, DDX5, CSTF1, SF3A1, SRSF6, SRSF2, PTK2, RBMX, BTRC, RAP1A, CTNNB1, and TSC2 (Figure 2(g)). Taking the intersection of hub genes and top module1, 11 genes were included and the other 8 genes were excluded, namely, UBA52, TSC2, SRSF2, RAP1A, PTK2, PTCH1, MAPK8, DDX5, CTNNB1, CDK6, and BTRC. Therefore, we selected these 11 genes as the key genes for this study.

### 3.3. Bioinformatics Analyses of Key Genes

**Figure 3:**
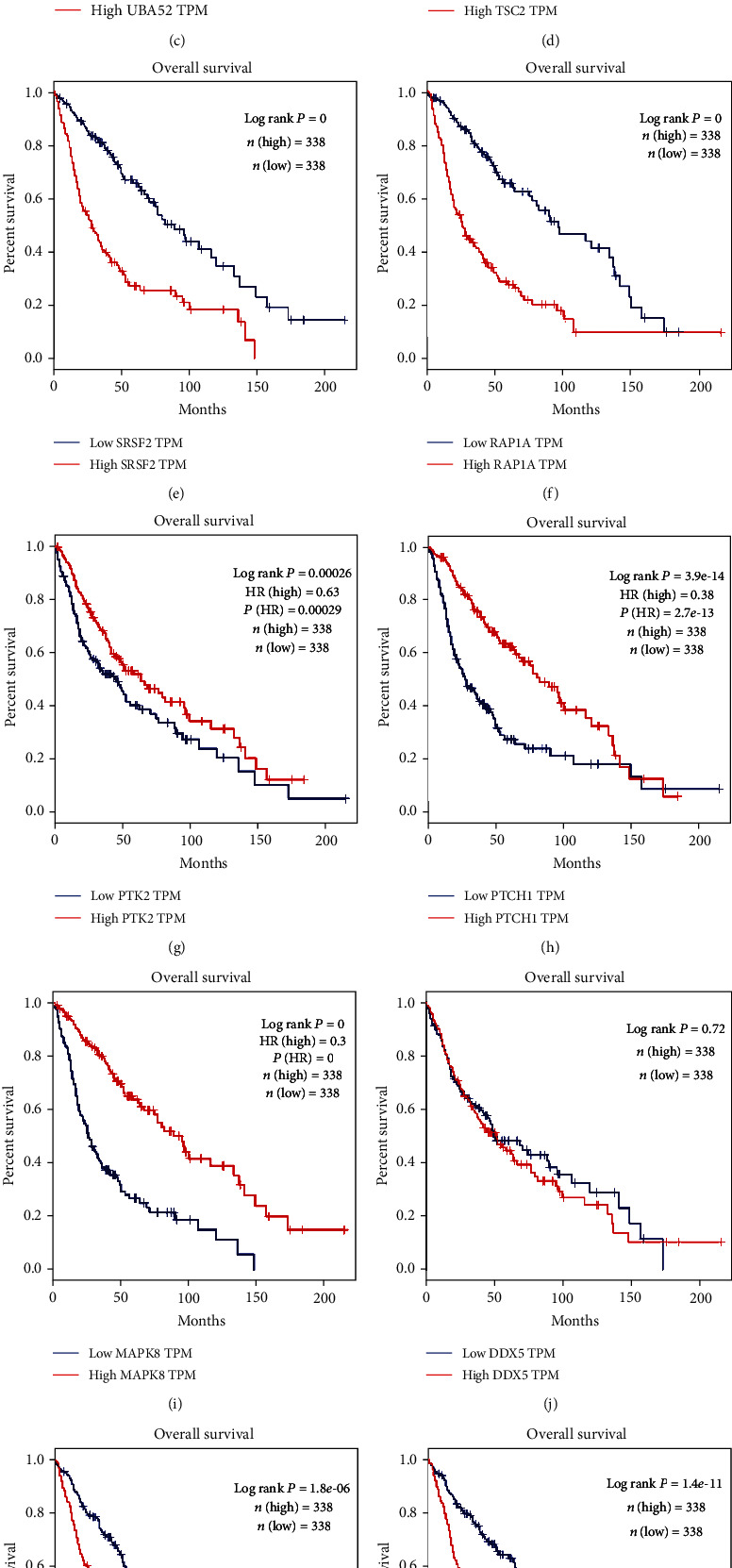
Bioinformatics analyses for key genes. (a) GO and (b) KEGG analyses for the 11 key genes. Kaplan–Meier survival curves of (c) UBA52, (d) TSC2, (e) SRSF2, (f) RAP1A, (g) PTK2, (h) PTCH1, (i) MAPK8, (j) DDX5, (k) CTNNB1, (l) CDK6, and (m) BTRC. The expression levels of (n) BTRC, (o) MAPK8, (p) PTCH1, (q) PTK2, and (r) TSC2 in GBM and normal tissues. ^∗^*P* < 0.05. Abbreviations: C: environmental information processing; CB: signal transduction; D: cellular processes; DA: transport and catabolism; DD: cellular community; E: organismal systems; EA: immune system; EF: nervous system; El: environmental adaptation; F: human diseases; FA: cancers: overview; FB: cancers: specific types; FG: endocrine and metabolic diseases; FH: infectious diseases: bacterial; FI: infectious diseases: viral; H: other and unknow; HA: other and unknow.

To further understand the 11 key genes, we performed GO and KEGG functional enrichment analyses and observed significant enrichment in various functional characteristics. As demonstrated in Figure 3(a), the GO enrichment analysis revealed that the 11 key genes in the biological process group were mainly associated with response to organic substance, cell surface receptor signaling pathway, and response to oxygen-containing compound. Cellular component analysis especially associated with the cytosol, neuron part, plasma membrane bounded cell projection, cell projection, perinuclear region of cytoplasm, especially neuron to neuron synapse. The GO enrichment analysis of molecular function was significantly enriched in protein binding, enzyme binding, drug binding, and cyclin binding. The results of KEGG analyses demonstrated that the key genes were mainly associated with pathway in cancer, focal adhesion, and cancer-related pathways including Wnt and cAMP pathway, Cushing syndrome, cellular senescence, Kaposi sarcoma-associated herpesvirus infection, human cytomegalovirus infection, and human papillomavirus infection (Figure 3(b)). In particular, neurotrophin signaling pathway was essential for the development of the vertebrate nervous system. Based on the survival analysis, 10 (UBA52 TSC2, CDK6, PTCH1, CTNNB1, PTK2, BTRC, MAPK8, SRSF2, and RAP1A) of the 11 key genes were closely related to the overall survival of patients with both glioblastoma multiforme (GBM) and low-grade glioma (LGG) (Figures 3(c)–3(m)). The overall survival analysis of DDX5 revealed that no significance was observed among high and low expressions of DDX5 patients (Figure 3(j)). Meanwhile, UBA52, SRSF2, RAP1A, CTNNB1, and CDK6 were associated with unfavorable prognosis of GBM and LGG patients (Figures 3(c), 3(e), 3(f), 3(k), and 3(l)). However, TSC2, PTCH1, MAPK8, PTK2, and BTRC had favorable outcomes of GBM and LGG patients (Figures 3(d), 3(h), 3(i), and 3(m)). Then, the expression levels of the 5 genes with good prognosis between GBM tissues and adjacent normal tissues were analyzed, indicated that expression of BTRC was lower in GBM tissues than in normal (Figure 3(n)). However, no statistically significant differences for MAPK8, PTCH1, PTK2, and TSC2 were observed (Figures 3(o)–3(r)). Collectively, these results demonstrated that BTRC expression was decreased in glioma tissues, and its expression levels were associated with a favorable prognosis of patients with glioma.

### 3.4. Upregulation of miR-193a-3p Promoted the Malignant Behavior of Glioma Cells

**Figure 4:**
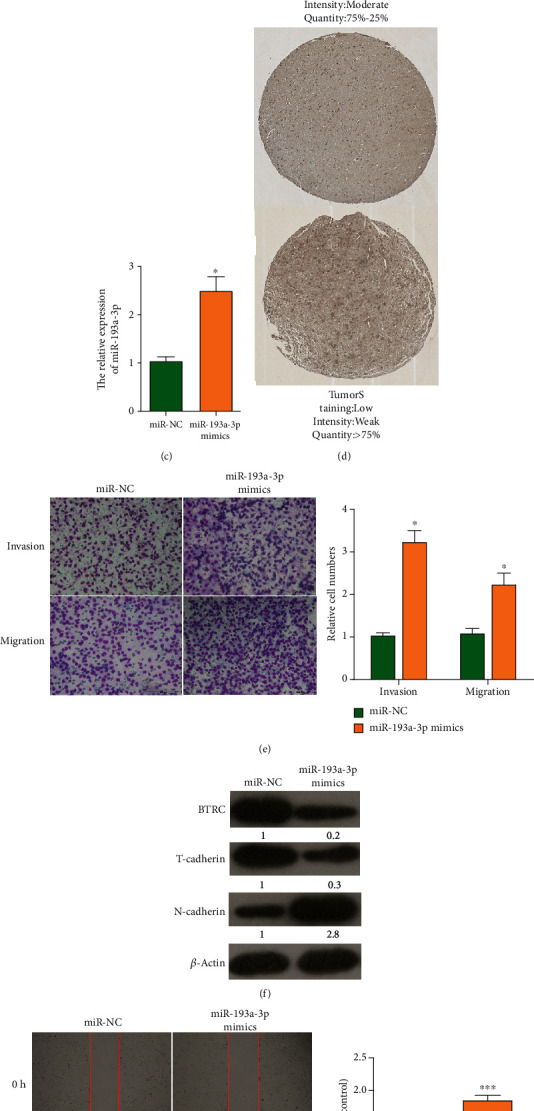
Effects of miR-193a-3p on the MT, migration, and invasion of glioma cells. (a) Expression level of miR-193a-3p in GSE26576. (b) Correlation curve between the expression level of miR-193a-3p and BTRC in glioma (*r* = −0.184 and *P* < 0.05). (c) Transfection efficiency of miR-193a-3p in LN-229 evaluated by qRT-PCR. (d) Expression of BTRC in normal glial cells and high-grade glioma tissues in HPA. (e) Invasion and migration abilities of LN-229 cells transfected with miR-NC and miR-193a-3p mimic groups analyzed by transwell assay. Scale bars, 100 *μ*m. (f) Western blot analyses for T-cadherin, N-cadherin, and BTRC in LN-229 cells transfected with miR-NC and miR-193a-3p mimics. (g) Migration capability of LN-229 cells transfected with NC and miR-193a-3p mimics analyzed by wound-healing assay at 0 and 24 h. Scale bars, 500 *μ*m. Data are shown as the means ± SD based on three independent experiments. ^∗^*P* < 0.05 and ^∗∗∗^*P* < 0.001.

To further investigate the expression of BTRC in glioma tissues, the mRNA data matrix of GSE26576 was downloaded from NCBI and the results exhibited a significantly lower BTRC in glioma tissues than in normal ones (Figure 4(a)). Starbase database was used to predict the relationship between miR-193a-3p and BTRC protein expression level in patients with glioma. Finally, it was confirmed that there was a significant negative correlation between the levels of BTRC and miR-193a-3p (Figure 4(b)). To investigate the potential role of miR-193a-3p in glioma, its mimic plasmids were transfected into LN-229 cells. qRT-PCR data showed that the relative expression of miR-193a-3p was significantly upregulated in miR-193a-3p mimic group, indicating a successful transfection (Figure 4(c)). Next, HPA database showed that the protein expression of BTRC was downregulated in glioma tumor tissues compared to normal ones (Figure 4(d)). These results confirmed our findings. Transwell assay showed that the migration and invasion abilities for LN-229 cells transfected with miR-193a-3p mimics were significantly upregulated compared to miR-NC (Figure 4(e)). We also detected the expression of N-cadherin, T-cadherin, and BTRC in groups of miR-NC and miR-193a-3p mimics by Western blot analysis. The results demonstrated a decreased expression of T-cadherin and BTRC but elevated N-cadherin in the miR-193a-3p mimic group, in comparison with the miR-NC group (Figure 4(f)). Moreover, wound-healing experiments showed that miR-193a-3p promoted cell migration (Figure 4(g)). These results indicated that miR-193a-3p should play an oncogenic role in glioma, and its elevation promoted the cell invasion, migration, and MT in glioma cell lines.

### 3.5. BTRC Was Involved in miR-193a-3p-Regulated Tumor Migration and Invasion Processes in Glioma Cells

**Figure 5:**
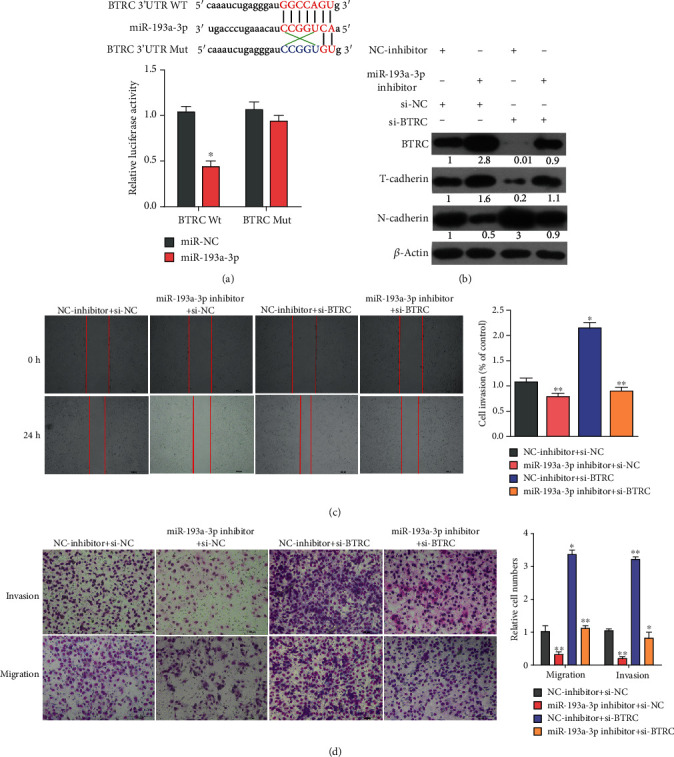
Effects of ectopic expression of miR-193a-3p and BTRC on the cell invasion, migration, and MT of glioma. (a) BTRC with WT 3′UTR or MUT 3′UTR was constructed into a luciferase reporter vector and cotransfected with miR-193a-3p mimics in LN-229 cells. Relative luciferase activity determined 48h after transfection. (b) LN-229 cells transfected with miR-NC, miR-193a-3p inhibitor, si-NC, and si-BTRC plasmid. The transwell assay was used to analyze the migration and invasion abilities of LN-229 cells in NC-inhibitor+si-NC, miR-193a-3p-inhibitor+si-NC, NC-inhibitor+si-BTRC, and miR-193a-3p-inhibitor+si-BTRC groups, and the cell number was statistically analyzed. Scale bars, 100*μ*m. (c) Migration ability in NC-inhibitor+si-NC, miR-193a-3p-inhibitor+si-NC, NC-inhibitor+si-BTRC, and miR-193a-3p-inhibitor+si-BTRC groups detected by wound-healing assay, and the migration distance was statistically analyzed. Scale bars, 500*μ*m. (d) Expression levels of BTRC, T-cadherin, and N-cadherin detected by Western blot. Data are shown as the means ± SD based on three independent experiments. ^∗^*P* < 0.05 and ^∗∗^*P* < 0.01.

Dual luciferase reporter assay confirmed the reduced luciferase activity after cotransfection of BTRC 3′UTR-WT and miR-193a-3p mimics compared to miR-NC and BTRC 3′UTR-MUT (Figure 5(a)). The results indicated that BTRC acted as a target gene for miR-193a-3p. Then, we explored the effects of miR-193a-3p on the invasion and migration of glioma cells by targeting BTRC through transwell and wound-healing assays. The results showed that miR-193a-3p-inhibitor+si-NC group inhibited the migration of LN-229 cells, with the results of NC-inhibitor+si-BTRC diametrically opposite, however. Meanwhile, miR-193a-3p-inhibitor+si-BTRC could reverse this phenomenon (Figures 5(b) and 5(c)). The expression level of N-cadherin was significantly decreased in the miR-193a-3p-inhibitor+si-NC group but significantly increased in the NC-inhibitor+si-BTRC group, which could be reversed by miR-193a-3p-inhibitor plus si-BTRC, while the expression level of T-cadherin was increased in the miR-193a-3p-inhibitor+si-NC group but decreased in the NC-inhibitor+si-BTRC group, which could be reversed by miR-193a-3p-inhibitor plus si-BTRC (Figure 5(d)). The results suggest that miR-193a-3p promotes malignant phenotypes of glioma cells in a BTRC-dependent manner.

## 4. Discussion

Glioma, also known as neuroectodermal or neuroepithelial tumor, is a disease with high mortality rate. According to the WHO, glioma accounts for about 75% of the malignant primary brain tumor [21]. The annual incidence of glioma in China is 3/60000, more prevalent in male patients than female ones. In addition, about 30,000 patients die from this disease each year [22]. Many miRNAs have been validated which were associated with the invasion and metastasis of glioma [23–25]. Since miRNAs can participate in complex biological functions in vivo, they can affect various biological processes and pathways through miRNA-mRNA regulatory networks [26, 27]. There has been increasing evidence for the vital role of miRNAs in the prognosis and treatment of many cancers. So far, a number of miRNAs with prognostic value, such as miR-196a [28], miR-503 [29], and miR-26b [30], have been proposed. Therefore, a comprehensive understanding of the role of miRNAs closely related to glioma can provide a new basis for finding sensitive specific miRNAs to glioma as biomarkers and searching for new therapeutic targets.

With the continuous development of biotechnology and innovation of new high-throughput technologies, genome-wide molecular level analysis has been proven to be an efficient method for identifying key genes involved in cancer development [31]. More and more researches have begun to investigate diseases at the genomic level using gene chip technology [32, 33]. In this study, the miRNA expression profiles of gliomas were retrieved from NCBI. The analysis suggests that miR-193a-3p is highly expressed in glioma, with elevated miR-193a-3p corresponding to poor prognosis. Its expression in glioma patients and cell lines was detected by qRT-PCR. The results were consistent with the microarray ones. The roles of miR-193a-3p in cancer progression have been reported that it inhibits the proliferation and migration of lung cancer and colorectal adenocarcinoma cells by targeting kirsten rat sarcoma viral oncogene (KRAS) [34, 35]. miR-193a-3p acts as a suppressor of metastatic disease progression in non-small-cell lung cancer (NSCLC) via the modulation of p53/Slug/L1CAM pathway [9]. Recently, many researchers have verified that miR-193a-3p acts as a tumor inhibitor. Takahashi et al. reported that miR-193a-3p is specifically downregulated and acts as a tumor suppressor in BRAF-mutated colorectal cancer [36]. miR-193a-3p could suppress proliferation and promote apoptosis by targeting cyclin D1 in hepatocellular carcinoma cells [37]. LncRNA of UCA1 functions as an oncogene in NSCLC, acting mechanistically by upregulating ERBB4 in part through the sponging of tumor suppressor miR-193a-3p [38]. In addition, miR-193a-3p was involved in the tumorigenicity of renal cell carcinoma (RCC) tissues and cell lines and can increase the proliferation and migration by targeting ST3GalIV via PI3K/Akt pathway in RCC cells [39]. Exosome-mediated transfer of miR-193a-3p from stromal cells to epithelial cancer ones contributes to cancer progression [40]. It was identified that silencing of miR-193a-3p through hypermethylation can promote HER2 positive breast cancer progress by targeting growth factor receptor bound protein 7 (GRB7), extracellular signal-regulated kinase 1/2 (ERK1/2), and forkhead box M1 (FOXM1) signaling [41]. Inhibition of miR-193a-3p reduced cell viability and increased the number of apoptotic gastric cancer cells [42]. Based on results from the literature, the expression levels of miR-193a-3p differed in the different tissues and organs, suggesting that different functions were required in different tissues. Further illustrated miR-193a-3p differently targets diverse systems and function by different mechanisms. In our study, we prove that miR-193a-3p was highly expressed in glioma, suppressing BTRC expression through directly targeting the 3′UTR of BTRC, suggesting the probable role of absent miR-193a-3p in the upregulation of BTRC. Nevertheless, further investigations are still needed to unravel the precise mechanism underlying the regulation of miR-193a-3p/BTRC axis in glioma.

Subsequently, we found 11 key genes from the PPI network. Each of these genes has been verified possessing important functions in cancer, with BTRC being the most noteworthy. This study has not only experimentally verified the role of miR-193a-3p in glioma, but suggested a network for miR-193a-3p to promote tumor progression through BTRC. BTRC belongs to a large family of F-box proteins that interact with phosphorylated substrates via WD40 repeats and recruit SCF*β*-TrCP E3 ubiquitin ligases to ubiquitinate and target them for degradation [43]. The modulation of BTRC level and activity plays an important role in several cancers [44]. In our study, the downregulated BTRC can obviously reverse the effects of miR-193a-3p inhibitor on the cell invasion, migration, and MT of glioma in vitro, suggesting BTRC functions as a tumor suppressor. Further studies are needed to elucidate the underlying mechanisms for this role of BTRC.

EMT was recognized as an important part for tumor invasion and migration processes. EMT participates in cancer development, tissue reformation, and organ fibrosis [45]. Recent studies suggest that prosaposin can regulate mesenchymal transition through the TGF-*β*1/Smad signaling pathway in GBM [46]. And miRNAs were reported involved in the regulation of epithelial phenotype and inhibition of EMT [47]. For example, miR-204-5p suppresses EMT and snail family transcriptional repressor 2 (STAT3) signaling pathways by targeting SNAI2, (SUZ12) polycomb repressive complex 2 subunit HDAC1, and Janus kinase 2 (JAK2) [48]. Upregulated miRNA-141 contributes to suppressing EMT and lymph node metastasis in laryngeal cancer through HOXC6-dependent TGF-*β* signaling pathway [49]. miR-205-3p has been reported a role in EMT process through inhibiting the expression of ZEB1 by binding the 3′UTR sites [50]. In this study, we proved that miR-193a-3p could bind to the 3′UTR of BTRC and its overexpression resulted in increased N-cadherin but decreased T-cadherin and BTRC in glioma cells. It was demonstrated that upregulated miR-193a-3p may downregulate BTRC and promote the MT process, which could be reversed by BTRC.

In conclusion, high expression of miR-193a-3p and low level of BTRC indicated worse overall survival for patients with glioma. And upregulated miR-193a-3p could promote the invasion, migration, and MT of glioma by downregulating BTRC. Overall, this study provides an understanding of causal biology research on glioma development. It provided a basis for miR-193a-3p and BTRC as biomarkers and therapeutic targets for glioma prognosis.

## 5. Conclusions

We reported that a specific miRNA called miR-193a-3p exhibited ectopic expression in glioma tissues and cells. The deregulation of miR-193a-3p contributed to a notable impairment of invasion, migration, and MT. Moreover, miR-193a-3p may act as a sponge to increase BTRC reduction. Our study reveals that high expression of miR-193a-3p and low level of BTRC indicated worse overall survival for patients with glioma. And upregulated miR-193a-3p could promote the invasion, migration, and MT of glioma by downregulating BTRC. Overall, this study provides an understanding of causal biology research on glioma development. It provided a basis for miR-193a-3p and BTRC as biomarkers and therapeutic targets for glioma prognosis.

## Figures and Tables

**Table 1:** Information of GEO datasets.

Accession No.	Platform	Organism	Gene or miRNA	Sample
GSE39486	GPL15829	Homo sapiens	miRNA	3 normal fetal brains and 3 glioma tissues
GSE26576	GPL570	Homo sapiens	Gene	35 glioma samples and 2 normal pediatric brainstem samples

**Table 2:** The clinical characteristic of CGGA patients.

Variables	Case, *N* (%)
Gender	
Male	123 (62.12%)
Female	75 (37.88%)
Type	
Primary	180 (90.90%)
Secondary	6 (3.00%)
Recurrent	12 (6.10%)
Grade	
WHO II	60 (30.30%)
WHO III	47 (23.74%)
WHO IV	91 (45.96%)

## Data Availability

All datasets generated for this study are included in the article.

## Abbreviations

MT: Mesenchymal transition
NCBI: National Center for Biotechnology
CGGA: Chinese Glioma Genome Atlas
GO: Gene Ontology
KEGG: Kyoto Encyclopedia of Genes and Genomes
PPI: Protein-protein interaction networks
MCODE: Molecular Complex Detection
GEPIA: Gene Expression Profile Interaction Analysis
BTRC: Beta-transducin repeat containing E3 ubiquitin protein ligase
miRNAs: MicroRNAs
qRT-PCR: Quantitative real-time PCR
HPA: Human Protein Atlas
GBM: Glioblastoma multiforme
LGG: Low-grade glioma
A: Metabolism
AH: Metabolism of cofactors and vitamins
B: Genetic information processing
BC: Folding, sorting, and degradation
C: Environmental information processing
CB: Signal transduction
D: Cellular processes
DA: Transport and catabolism
DC: Cell growth and death
DD: Cellular community
E: Organismal systems
EA: Immune system
EB: Endocrine system
EF: Nervous system
El: Environmental adaptation
F: Human diseases
FA: Cancers: overview
FB: Cancers: specific types
FH: Infectious diseases: bacterial
FI: Infectious diseases: viral
FG: Endocrine and metabolic diseases
H: Other and unknow
HA: Other and unknow.

## Supplementary Materials

Supplementary Materials Supplementary Figure 1A: Venn diagram showed the overlap of shared target genes for miR-193a-3p in miRWalk, DIANA, and starbase. B: PPI analysis for target genes.
