# Supplementary material for: miR-193a-3p Promotes the Invasion, Migration, and Mesenchymal Transition in Glioma through Regulating BTRC
Source: Biomed Res Int. 2021 Feb 9;2021:8928509. doi: 10.1155/2021/8928509 (PMC7886567; doi:10.1155/2021/8928509)
Supplement: Supplementary Materials — Supplementary Figure 1A: Venn diagram showed the overlap of shared target genes for miR-193a-3p in miRWalk, DIANA, and starbase. B: PPI analysis for target genes. [file 8928509.f1.docx]

## Supplementary Materials


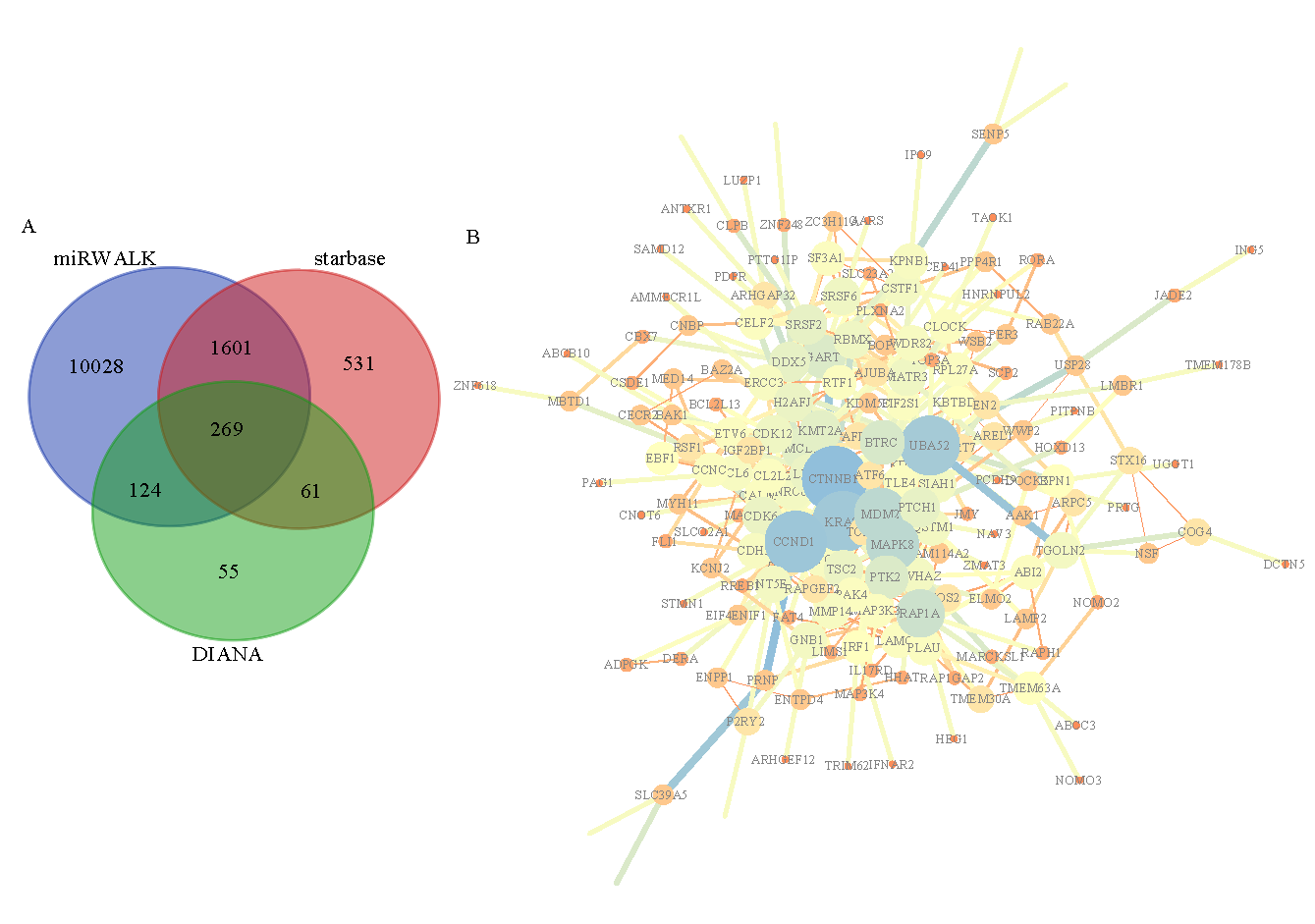


Supplementary Figure 1A. Venn diagram showed the overlap of shared target genes for miR-193a-3p in miRWALK, DIANA, and starbase. B PPI analysis for target genes.
